# Commentary: Do Thirty-Second Post-activation Potentiation Exercises Improve the 50-m Freestyle Sprint Performance in Adolescent Swimmers?

**DOI:** 10.3389/fphys.2019.00215

**Published:** 2019-03-18

**Authors:** José Afonso, Cosme F. Buzzachera, Ricardo J. Fernandes

**Affiliations:** ^1^Faculty of Sport, Centre of Research, Education, Innovation and Intervention in Sport, University of Porto, Porto, Portugal; ^2^Department of Physical Education, North University of Paraná, Londrina, Brazil; ^3^Porto Biomechanics Laboratory, University of Porto, Porto, Portugal

**Keywords:** post-activation potentiation, adolescents, strength, swimming, dry-land training

The intent of this commentary is to constructively contribute to the recently published manuscript by Abbes et al. (2018) by debating some of the referred topics. We believe the authors have delivered a compelling case and presented a solid methodology, contributing significantly to the swimming related body of knowledge. In fact, post-activation potentiation (PAP) is a topic of growing interest in both power and endurance sports (cf. Boullosa et al., 2018) and its practical application in swimming, even if some approaches are already available (e.g., Barbosa et al., 2016; Cuenca-Fernández et al., 2018), is lacking of scientific insights. Therefore, aiming to find out if PAP re-warm up helps increasing adolescent swimmers competitive performance is of substantial relevance. Furthermore, the authors were very careful and thoughtful in their line of reasoning and especially when analyzing their data. Notwithstanding, we aim to provide additional insights that may expand the discussion on the subject, calling attention to a scholarly paper of particular note and to a topic of great interest. Table 1 summarizes some suggestions for improving the methodologic approach.

**Table 1:** Suggestions for improving methodologic approach.

1.	When applying exercises that are highly influenced by body weight (BW), group analysis according to subjects' BW.
2.	The balance may have favored fatigue instead of PAP. It would be interesting to experiment with shorter warm-ups and longer and/or more intense PAP protocols.
3.	Perhaps the exercises were not so specific enough, and therefore the neural pathways specific for the competition were not sufficiently stimulated.
4.	When analyzing data, perhaps a focus on interindividual variation would provide a different reading of the results.

Abbes et al. (2018) have selected a sample with swimmers in a 4-year range in ages (i.e., 11 to 15 years-old). Even though the authors argued, supported by evidence, that age may not play a factor in the mechanisms of PAP, in this case such age difference translated into a considerable variation in body weight (BW), with a standard deviation of ± 9.5 kg of an average of 52.5 kg, representing roughly 20% of BW. This becomes relevant as the exercises used in the three PAP protocols involved overcoming the BW, and biomechanics can be changed significantly by only 10% changes in body mass (Kulas et al., 2008). Therefore, it is highly likely that each participant felt a very different percentage of maximal load. This factor may have produced diversified physiological responses which, when averaged-out, resulted in an apparent lack of potentiation effect.

Another relevant issue concerns the balance between PAP and fatigue, which was widely discussed by the authors (both in the introduction and in the discussion sections). Considering that the initial warm-up was a standard swimming workout (cf. Neiva et al., 2015), perhaps this load had a too large magnitude in comparison to the 30 s PAP protocols. In fact, the 1,200 m distance was proposed as the preferential warm-up but for older swimmers (18.1 ± 3.3 years old), who were of a higher competitive level comparing to the current ones. If that is the case, then the mechanisms involving fatigue would likely surpass any benefits from PAP protocols. Alternatively, maybe such a prolonged warm-up (especially considering the nature of the competitive distance) brings such fatigue levels that the addition of PAP protocols in that timing are inducing more additional fatigue than an effective potentiation (Boullosa et al., 2018). Overall, with a shorter warm-up [as the alternative one proposed by Neiva et al. (2015)], followed by a more prolonged and/or intense PAP workout, perhaps the desired potentiation effect would have been achieved.

In swimming in particular, for potentiating performance, it is fundamental that muscular strength increments translate into increased ability to generate propulsive force in water, with technical aspects of swimming mechanics determining the extent to which increased force transfers into faster swimming velocity (Girold et al., 2007; Mujika and Crowley, 2018). Therefore, we disagree with Abbes et al. (2018) that push-ups, squat jumps, and burpees are swimming specific dry-land exercises, even if the same muscles used in front crawl (not in freestyle, as this is a swimming event, not a swimming technique as the authors erroneously wrote) are stimulated. In fact, sprinting velocity is closely dependent on coordination and technique (Cronin et al., 2007) and, in front crawl, wrist flexion, elevation of the elbow, longitudinal axis body rotation, and lower limbs internal rotation are critical points (Maglischo et al., 1988; Sanders and Psycharakis, 2009) not found in any of those dry-land exercises. Knowing that muscle strength is not a whole-body characteristic but is dependent on the ability and trainability of specific body segments to perform the desired movement (Pearson et al., 2006; Nikolaidis, 2012), probably the in-water PAP routines (e.g., with paddles, fins, or parachutes) would result better, even knowing that they are hard to accomplish due to competitive facilities and rules constraints.

The role of inter-individual variation in response to any training load should also be analyzed (Chen et al., 2017). Even if the average response of a given sample or group denotes a lack of effect of an intervention protocol, that may be due to distinct responses by each individual, which, when grouped and averaged-out, result in an apparent lack of effect (Dunne et al., 2012). It is possible that certain subgroups of participants have benefited from the intervention, while others have seen their performance impaired. An analysis of the response of each individual or subgroup would perhaps shed a different light and complement the data concerning average group responses. Indeed, the authors state that individualization of PAP protocols should be aimed at (cf. Table 1).

Finally, we agree with Abbes et al. (2018) in what concerns the importance of the warm-up period prior to the race event. However, those authors, as the swimming science community in general, seem to oversize the importance of the physiological activation, neglecting the relevance of the swimmers adaptation to a new context (Seifert et al., 2007; Pyne and Sharp, 2014). In fact, every single coach will support the importance of allowing young and adolescent swimmers getting used to different lighting conditions, colder or hotter water temperatures, rough or slippery starting blocks and turning walls surfaces (as well as specific handgrips and wedges for backstroke starts), specific acoustics (including the relevant “take your marks and start signal” command voice) and visual signaling (suspended flagged ropes 5 and 15 m from each end wall, floats of different colors in the lane ropes and distinctive marks placed on the floor of the pool in the center of each lane) (cf. Tipton and Bradford, 2014; de Jesus et al., 2016; Guignard et al., 2017). This is, probably, equally (or more) important for a good performance than PAP exercises. At least at non-elite level where swimmers are not so much at ease with diverse (and stressful) competitive environments.

## Author Contributions

JA, CB, and RF have fully reviewed and criticized the original article, drafted the commentary, reviewed, and approved the final manuscript.

### Conflict of Interest Statement

The authors declare that the research was conducted in the absence of any commercial or financial relationships that could be construed as a potential conflict of interest.
